# Exogenous TSG-6 treatment alleviates DSS-induced colitis in mice by modulating Pou2f3 and promoting tuft cells differentiation

**DOI:** 10.1186/s10020-025-01230-5

**Published:** 2025-04-29

**Authors:** Shaopeng Yang, Yuqi Li, Rongwei Ruan, Jiangping Yu, Bo Zhu, Haibin Lou, Xiaolan Zhang, Shi Wang

**Affiliations:** 1 Department of Endoscopy, Zhejiang Cancer Hospital, Hangzhou Institute of Medicine (HIM), Chinese Academy of Sciences, Hangzhou, Zhejiang 310022 China; 2 Department of Gastroenterology, The Second Hospital of Hebei Medical University, Shijiazhuang, Hebei 050000 China

**Keywords:** Tumor necrosis factor α stimulated gene 6, Inflammatory bowel disease, Intestinal barrier, Tuft cells, Pou class 2 homeobox 3

## Abstract

**Background:**

Whereas intestinal epithelial barrier dysfunction is implicated in inflammatory bowel disease (IBD), the underlying mechanisms remain elusive. Tumor necrosis factor α stimulated gene 6 (TSG-6) is a secretory protein with anti-inflammatory properties. Our previous research demonstrated TSG-6 can relieve intestinal inflammation and mucosal damage. However, the underlying mechanism and targets remain unclear. This research sought to explore how TSG-6 regulates the intestinal epithelial barrier and its mechanistic role in experimental colitis.

**Methods:**

IBD mouse model was generated using dextran sodium sulfate (DSS), with or without intraperitoneal injection of TSG-6(100 µg/kg or 200 µg/kg). The effects of TSG-6 on colonic inflammation and intestinal barrier function were investigated. Label-free quantitative proteomic analysis was performed on intestinal samples to explore the mechanism and therapeutic target of TSG-6. Molecular interactions were determined by co-immunoprecipitation (Co-IP) and immunofluorescence colocalization.

**Results:**

TSG-6 treatment significantly attenuated DSS-induced colitis symptoms and inflammatory cell infiltration. Microarray analysis revealed that TSG-6 decreased pro-inflammatory cytokine levels in colon tissue. TSG-6 restored the intestinal epithelial barrier through the promotion of intestinal epithelial cells (IECs) proliferation and mitigation of tight junctions (TJs) damage. Mechanistically, TSG-6 promoted tuft cells differentiation and increased interleukin-25 (IL-25) levels by directly binding to Pou class 2 homeobox 3(Pou2f3) and up-regulating its expression in the gut.

**Conclusions:**

This study demonstrated TSG-6 as a positive regulator of tuft cells differentiation by interacting with Pou2f3, and the effectiveness of exogenous TSG-6 treatment on maintaining intestinal barrier integrity showed a promising potential for its clinical application.

**Supplementary Information:**

The online version contains supplementary material available at 10.1186/s10020-025-01230-5.

## Introduction

Inflammatory bowel disease (IBD), encompassing ulcerative colitis (UC) and Crohn’s disease (CD), represents a long-lasting and relapsing–remitting illness with globally escalating prevalence, particularly across numerous developing nations, posing a notable economic and healthcare challenge (Celiberto et al. 2018; Kaplan 2015; Kaplan and Windsor 2021). Despite decades of research, the etiology of IBD remains unclear, and existing therapeutic strategies continue to face limitations in both diagnostic accuracy and treatment efficacy, underscoring the urgent need for novel therapeutic approaches.

The integrity of the intestinal epithelial barrier is crucial for maintaining intestinal homeostasis, and epithelial barrier dysfunction is a momentous feature of IBD (Zha et al. 2023). Preserving intestinal epithelial integrity effectively alleviated intestinal inflammation in IBD animal models (Bai et al. 2020). This dynamic barrier system comprises intricately organized intestinal epithelial cells (IECs) interconnected by tight junctions (TJs), collectively forming the frontline defense against luminal pathogens and macromolecules (Peterson and Artis 2014). Intestinal stem cells (ISCs) orchestrate epithelial regeneration through coordinated proliferation and differentiation into specialized IEC subtypes upon injury signals, though the specific IEC populations critical for mucosal repair remain poorly defined (Leung et al. 2018).

The intestinal epithelium is mainly composed of enterocytes for absorbing nutrients; goblet cells for mucus production; paneth cells, responsible for generating antimicrobial peptides; and enteroendocrine cells that secrete regulatory hormones (Stephens and Weid 2020). Recent advances have identified tuft cells as pivotal mediators of epithelial restoration, characterized by their signature markers Double cortin-like kinase 1 (Dclk-1) and the master transcriptional regulator Pou2f3 (Westphalen et al. 2014; Gerbe et al. 2012; Sato et al. 1989). These chemosensory cells execute dual barrier-enhancing functions through: (1) secretion of cysteinyl leukotrienes (cysLTs) to mobilize type 2 immunity against helminthic threats (McGinty et al. 2020a), and (2) production of IL-25 that amplifies epithelial defenses via downstream effectors including IL-4, IL-13, and secretory phospholipase A2 (sPLA2) (Heneghan et al. 2014). Emerging evidence positions tuft cells as sentinel regulators limiting bacterial translocation while accelerating epithelial repair mechanisms (Yi et al. 2019), making them attractive therapeutic targets for IBD intervention.

Tumor necrosis factor-stimulated gene-6 (TSG-6) is a secreted protein known for its tissue protective and immune regulatory functions (Day and Milner 2019). Past research has confirmed its remarkable tissue-protective capacities in corneal and dermal wound healing models through mesenchymal stem cell (MSCs) -mediated mechanisms (Al-Jaibaji et al. 2020; Qi et al. 2014). Our previous research showed that exogenous injection of TSG-6 can effectively relieve intestinal inflammation and elevate the goblet cell count in mice with colitis (Yang et al. 2021), yet its precise impact on epithelial barrier restoration remains unclear. Given the differential regulation of IEC subtypes during mucosal repair (Clevers 2013; Ayyaz et al. 2019; Durand et al. 2012), we hypothesized that TSG-6 might preferentially modulate tuft cell-mediated regenerative pathways. In this study, we integrate proteomic profiling with functional validation to elucidate TSG-6's novel role in promoting tuft cell differentiation and subsequent mucosal healing. Our findings establish TSG-6 as a potent regulator of intestinal barrier homeostasis through epithelial reconstitution mechanisms, providing new therapeutic avenues for IBD management.

## Materials and methods

### Experimental animals

Specific pathogen-free male C57BL/6 mice (6–8 weeks old, 18–22 g) were purchased from Beijing Vital River Laboratory Animal Technology Co. Ltd. Throughout the duration of the experiment, the mice had free access to water and food within a controlled setting, which was maintained at a temperature of 24 ± 2℃, humidity levels of 55 ± 10%, and a consistent 12-h light/dark cycle. The conduct of animal experiments was approved by the Welfare and Ethical Committee for Experimental Animal Care of Zhejiang Cancer Hospital, adhering to rigorous guidelines for the management and ethical treatment of experimental animals (experimental animal ethics number zjzlsd-2023–02–057).

### Human samples

Human colonic biopsies were collected by Zhejiang Cancer Hospital, Hangzhou, China. The diagnosis of UC was based on evaluations through colonoscopy and pathological analyses of colonic biopsy specimens. Normal human colon tissue was used as healthy control, which were obtained from the patient without a recent or chronic illness. The procedure for consenting received approval from the ethics committee, and all participants provided written informed consent before tissue samples collection (ethics approval number IRB-2023–1017).

### Colitis animal model

2.0% DSS (molecular weight, 36,000 to 50,000Da; catalog 0216011080, MP Biomedicals, Irvine, CA, USA) was dissolved in drinking water and was orally administered to male C57BL/6 mice for 7 days to establish chemically-induced colitis model, while control mice (6 mice per group) received water without DSS. To evaluate the therapeutic effect of TSG-6 in DSS-induced colitis model, recombinant human TSG-6 protein (rhTSG-6) (catalog 2104-TS-050, R&D, Minneapolis, MN, USA) was injected intraperitoneally (day 5–9, daily) at a dose of 200 µg/kg in DSS + TSG-6 high dose group (DSS + H-TSG-6, *n* = 6), and mice in DSS + TSG-6 low-dose group (DSS + L-TSG-6, *n* = 6) received 100 µg/kg TSG-6 at the same time, following the protocol described in previous studies (Yang et al. 2021; Sala et al. 2015). DSS + PBS group (*n* = 6) received equal volume of PBS solution administration.

### Assessment of colitis

The mice in the study were observed and evaluated for disease severity on a daily basis using the disease activity index (DAI) numerical system, which has been previously described (Wirtz et al. 2017). Colon tissue samples were gathered and preserved in formaldehyde prior to staining with hematoxylin and eosin (H&E). The degree of inflammation in the mice model was evaluated by histopathological damage analysis using the Cooper HS score system (Cooper et al. 1993).

### Intestinal permeability assay

The intestinal epithelium permeability of mice was assessed on the 10 th day following DSS treatment, which was conducted by administering fluorescein isothiocyanate-dextran (FITC-D, 4 kDa; catalog 46,944,Sigma‐Aldrich, St. Louis, USA). To examine the content of FITC-D in blood, the mice underwent a 4-h fast before being administered a FITC-D solution (60 mg/100 g) through oral gavage. Four hours post-administration, blood was collected via retro-orbital bleeding under anesthesia. After that, the mouse was euthanized by overdose anesthetic (sodium pentobarbital). The serum was obtained by centrifuged at 12,000 g for 5 min. The concentration of FITC-D was measured by fluorescence spectrophotometer (excitation 490 nm/emission 520 nm). Additionally, the intestine tissue was preserved in OCT compound, sliced into 5-μm sections, stained with 4’,6-diamidino-2-phenylindole (DAPI, blue), and analyzed using a fluorescence microscope to visualize the distribution of FITC-D (green) inside the gut.

### Immunofluorescence staining

Immunofluorescence analysis was used to determine the intestinal epithelial distribution of the tight junction proteins and Tuft cell, including Zonula Occludens 1 (Zo-1), Claudin-1, Occludin and Dclk-1. The process of antigen retrieval included boiling the paraffin sections, which had been deparaffinized and rehydrated, in a pressure cooker for 6 min in total with Tris–EDTA buffer (10 mM Tris base, 1 mM EDTA, pH 9.0). Sections were blocked with 3% bovine serum albumin and incubated with primary antibody Zo-1(catalog ab221547, 1:100 dilution, Abcam, Cambridge, UK), Occludin (catalog ab216327, 1:100 dilution, Abcam, Cambridge, UK), Claudin-1(catalog ab211737, 1:500 dilution, Abcam, Cambridge, UK), or Dclk-1 (catalog ab31704, 5 µg/ml concentration, Abcam, Cambridge, UK) overnight at 4 ^◦^C. The sections were washed with PBS three times and then incubated with a secondary antibody (goat anti-rabbit, 1:1000 dilution, Life Technologies). Finally, those sections were counterstained with DAPI for 1 h at room temperature. The Olympus confocal laser scanning microscope (FV3000, Tokyo, Japan) was used to visualize the slides. The procedure for confocal fluorescence microscopy is described in the Supplementary Materials and Methods. The colocalization of TSG-6 and Pou2f3 was analyzed by image J.

### Immunohistochemical analysis

Colon tissue from either a human or mouse was initially fixed using 4% paraformaldehyde, encased in paraffin and finely sliced into 4 µm sections. Prior to subjecting the paraffin sections to antigen repair using a 3% citric acid solution, the paraffin had to be dewaxed. In order to block non-specific binding of antigens, a 10% goat serum was applied before the addition of the primary antibody. The following primary antibodies were used: myeloperoxidase (MPO, catalog ab300650, 1:5000 dilution, Abcam, Cambridge, MA), ki-67 (catalog GB151142-100, 1:500 dilution, Servicebio, Wuhan, China), and Dclk-1(catalog ab109029, 1:100 dilution, Abcam, Cambridge, UK). Images were captured using a light microscope with magnifications of 100x, 200x, or 400x. The expression of the target protein was quantified with ImageJ, following the methodology outlined in the previous study (Garrity-Park et al. 2012).

### Periodic Acid‑Schiff (PAS) staining

After deparaffinization, PAS staining was performed using the staining kit (catalog G1049-20ML, Servicebio, Wuhan, China). Briefly, the sections were treated with 0.5% periodic acid and placed in Schiff's reagent. Next, the samples were counterstained with a modified Gill III hematoxylin solution, followed by dehydration and then mounted with Toluene Permount Mounting Medium.

### Western blot

Western blot was performed as detailed previously (Yang et al. 2021). Colon tissue samples from control group, DSS + PBS group and DSS + H-TSG-6 group were detected with primary antibodies against Pou2f3 (catalog BS74340, 1:100 dilution, Bioworld Technology, Nanjing, China). Densitometric analysis was conducted via Image J software to determine the content of Pou2f3 protein, which were then normalized to β-actin.

### Molecular docking assay

The protein structure of TSG-6 used for docking was obtained from PDB database (https://www.rcsb.org/). Pou2f3 mRNA sequence was downloaded from (https://www.ncbi.nlm.nih.gov/) after retrieval, and the protein structure models of Pou2f3 was built using ADF2 software https://colab.research.google.com/github/sokrypton/ColabFold/blob/main/AlphaFold2.ipynb). GalaxyWEB (https://galaxy.seoklab.org/c) was used to perform protein docking analysis between structure of TSG-6 and Pou2f3. Protein structural visualization and interaction analysis were performed in PyMOL software (http://www.pymol.org).

### Cytokine gene microarray

The qPCR Array for Mouse Cytokines and Chemokines was utilized to determine gene expression profiles adhering to the manufacturer's instructions (Wcgene Biotech, Shanghai, China). WCgene Biotech software was utilized to analyze the acquired data. Genes exhibiting fold change greater or less than 2.0 were considered biologically significant.

### Transmission electron microscopy (TEM)

Fresh colon tissue was fixed using TEM fixative at 4 °C. Subsequently, the tissue was dehydrated with ethanol at ambient temperature and embedded in resin. Cut the resin block into 60-80nm thin pieces and stain with uranyl acetate and lead citrate for 8 min. Images were captured with a transmission electron microscope (H7800; Hitachi Ltd., Japan).

### Label-free quantification proteomics

Specific steps are described in detail in the supplementary material. Briefly, 20 μg proteins extracted from each group was loaded onto the 12% SDS-PAGE gel and run into the gel at approximately 2 cm. Protein bands were examined via Coomassie Blue R-250 staining after electrophoresis. Proteins were precipitated using acetone, then resuspended and mixed with trypsin buffer, and the resulting peptides were collected as a filtrate. For label-free quantification, free academic software Maxquant was implemented. Significantly differentially expressed proteins were identified by fold change > 1.5 and *P* < 0.05.

### Statistical analysis

Data sets were represented as the mean ± standard deviation (SD). Unpaired Student’s t-test was utilized to test difference between two groups. One-way analysis of variance (ANOVA) with Tukey’s multiple comparison tests was employed for comparisons across multiple groups. Immunofluorescence colocalization analysis was performed by the Pearson correlation coefficient using ImageJ software. *P* < 0.05 was considered as statistically significant. Statistical analysis was performed using GraphPad Prism version 8.0 for Windows (GraphPad Software, San Diego, California USA).

### Bioinformatics analysis

Gene ontology (GO) and Kyoto Encyclopedia of Genes and Genomes (KEGG) were utilized to analyze the functional information of the identified proteins. The Animal Transcription Factor Database (AnimalTFDB 3.0) was employed for annotating and predicting animal transcription factors (TFs). For a more comprehensive understanding of the methods used, please consult the supplementary material.

## Results

### TSG-6 attenuates DSS-induced colitis symptoms in mice

**Fig. 1:**
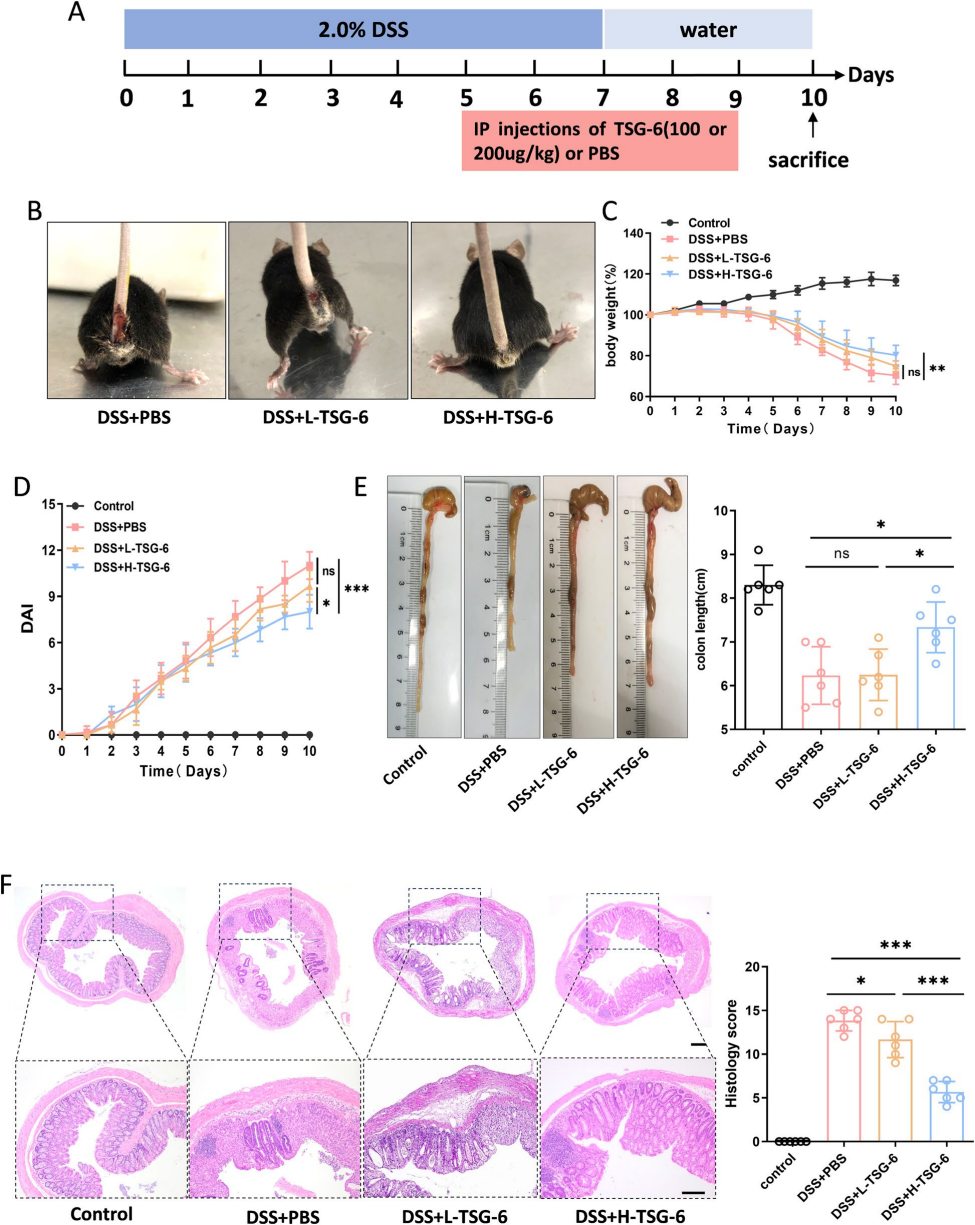
TSG-6 attenuates DSS-induced experimental colitis. **A** Schematic representation of experimental design. Mice were administered with 2.0% DSS for 7 days to induce colitis. Recombinant human TSG-6 was injected intraperitoneally (100 or 200 µg/kg, daily from day 5 to day 9). **B** Hematochezia in the DSS + PBS and DSS + L-TSG-6 mice was more serious than mice of DSS + H-TSG-6 on day 10. **C**, **D** Body weight and DAI of mice were monitored and recorded daily as an indirect measure of colitis severity. **E**, **F** Colon length, HE staining and histological score in the control, DSS + PBS, DSS + L-TSG-6 and DSS + H-TSG-6 groups at day 10(*n* = 6). Scale bar, 200 μm. Data are presented as mean ± SD. *P < 0.05, **P < 0.01, ***P < 0.001 and ns indicates* P* > 0.05

Mice received DSS treatment for 7 days with or without TSG-6 to evaluate efficacy of TSG-6 on colitis. The mice were randomly divided into four groups: Control, DSS + PBS, DSS + L-TSG-6(100 µg/kg, i.p.), and DSS + H-TSG-6(200 µg/kg, i.p.). Most mice in the DSS + PBS group presented sticky and bloody stools after 7 days of DSS treatment, with this condition persisting through day 10. In contrast, mice administered TSG-6 (200 µg/kg) initially had bloody stools that subsequently resolved (Fig. 1B). As shown in Fig. 1C, body weight loss can be easily observed in DSS + PBS group. Conversely, a dose-dependent attenuation of weight loss was noted in colitis mice following intraperitoneal injection of TSG-6. Moreover, mice receiving a higher dose of TSG-6 exhibited longer length of colon and decreased DAI score (Fig. 1D, E). H&E staining shows obvious inflammatory cell infiltration and distortion of crypts in the colon tissues after DSS treatment, and TSG-6 attenuated histologic inflammation and protected the structure of colon crypt in a dose-dependent manner (Fig. 1F). Our data indicate that TSG-6 attenuated DSS-induced colitis symptoms in a dose-dependent manner.

### TSG-6 reduces inflammatory cytokines expression and MPO levels

**Fig. 2:**
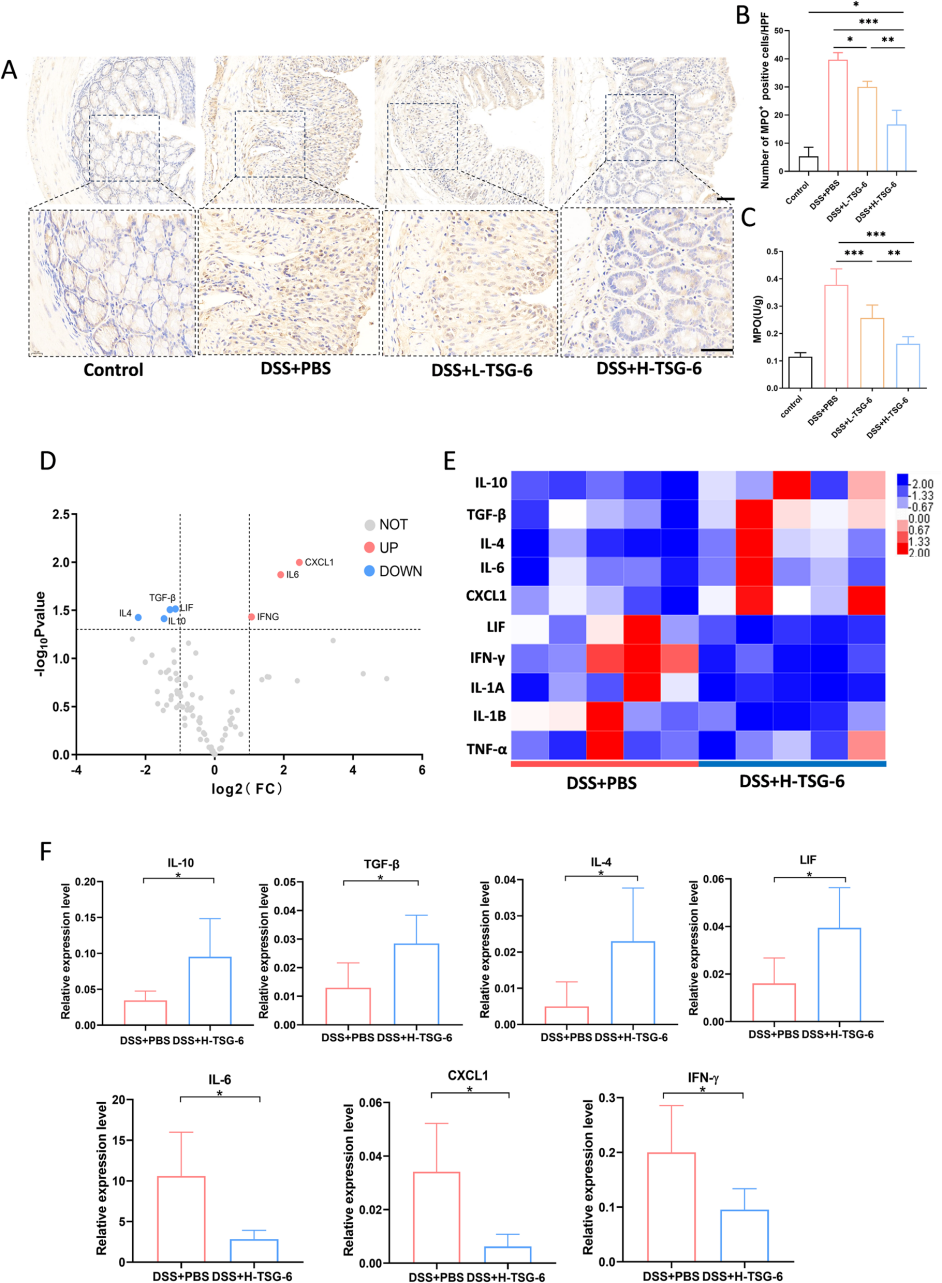
Treatment of TSG-6 reduces MPO levels and altered cytokine profile. **A** Immunohistochemistry showing the MPO positive cells in colon tissue. Scale bar, 50 μm. **B** Quantification of positive cells per high power field (HPF). **C** MPO activity in colon was quantified by ELISA. **D** Volcano plot showing mRNA with differential expression in DSS + PBS group and DSS + H-TSG-6 group. **E** Hierarchical clustering of representative cytokine mRNA levels between two groups. In the heatmap, high expression was shown in red, and low expression was shown in blue. **F** qPCR results showing the mRNA levels of IL-10, TGF-β, IL-4, LIF, IL-6, CXCL1, IFN-γ in two groups(*n* = 5). Data are presented as mean ± SD. *P < 0.05, **P < 0.01 and ***P < 0.001

To investigate neutrophils infiltration in colon, we performed immunohistochemistry and activity assay for MPO, a specific enzyme expressed by neutrophils. Results shown in Fig. 2A-C indicate that the count of MPO-positive cells and MPO activity in colon tissue significantly increased in DSS + PBS group as compared to DSS + L-TSG-6 and DSS + H-TSG-6 group, and higher dose of TSG-6 treatment significantly reduced MPO-positive neutrophils infiltration and its activity. To further investigate changes of cytokines caused by TSG-6 in the colitis mice, we performed a cytokines and chemokines qPCR Array, including 90 inflammation-related genes. Cluster analysis heat map and volcano plot were shown to present the differentially expressed genes (DEGs) (Fig. 2D, E). The heatmap displays elevated expression levels of DEGs in red and reduced levels in blue. qPCR analysis demonstrates that the higher dose administration of TSG-6 upregulated the levels of anti-inflammatory factors such as leukemia inhibitory factor (LIF), IL-4, IL-10, and transforming growth factor-beta (TGF-β) and inhibits the expression of pro-inflammatory markers, including chemokine ligand 1 (CXCL1), IL-6, and interferon-gamma (IFN-γ), compared to the DSS + PBS group (Fig. 2F).

### TSG-6 reverses the epithelial barrier damage in colitis mice

**Fig. 3:**
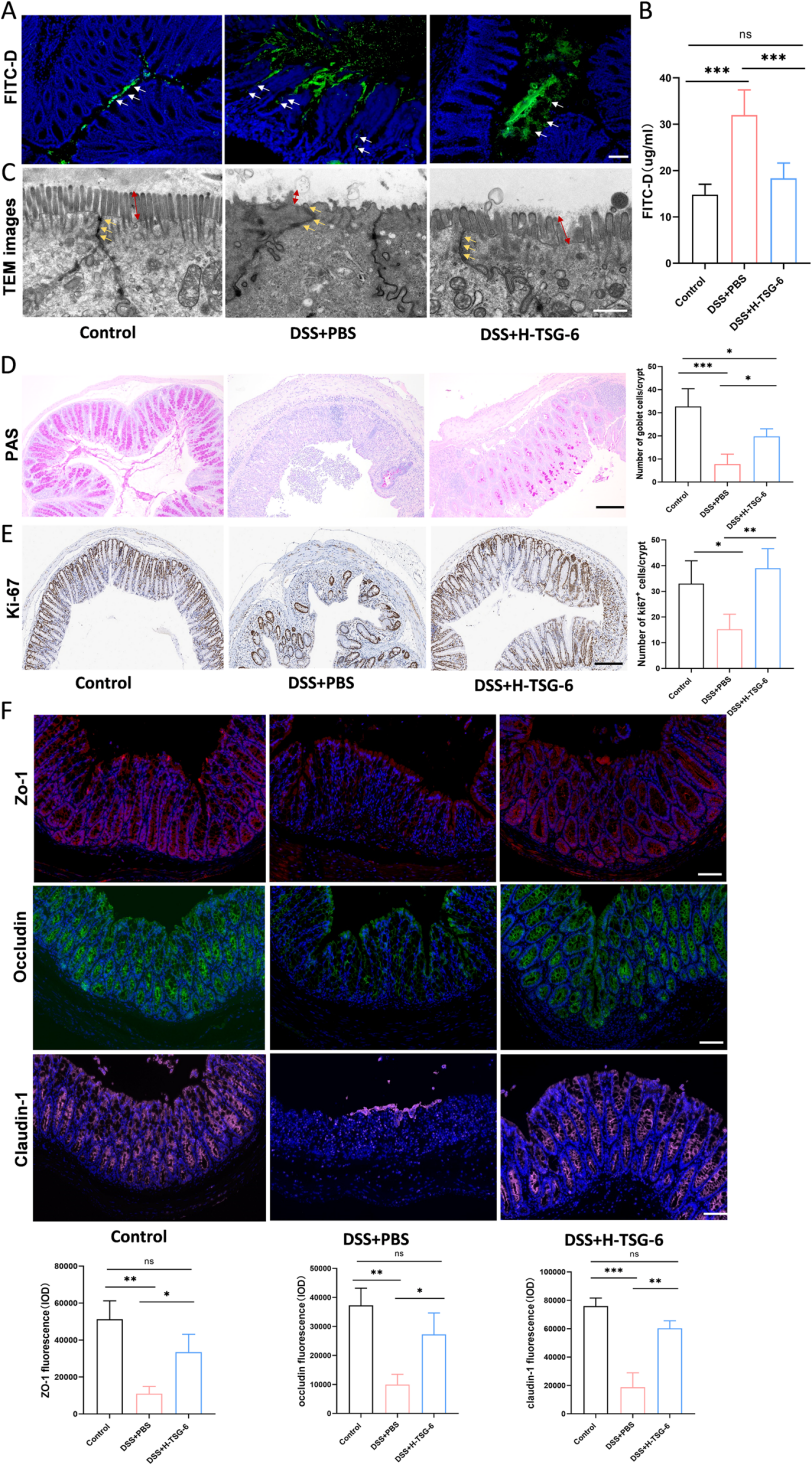
TSG-6 restores the integrity of intestinal epithelial barrier in colitis mice. **A** The distribution of FITC-D in intestinal tissue under fluorescence microscope. DAPI located in the nucleus (blue), and green represents FITC-D (white arrow). Scale bar, 50 μm. **B** FITC-D concentration in serum indicative of gut permeability. **C** The ultrastructural morphology of tight junctions (yellow arrows) and microvilli (red arrow) were observed under transmission electron microscope (TEM). Scale bar, 1 μm. **D**, **E** PAS staining for goblet cells and immunohistochemical staining of Ki67 in colon sections. Quantification of positive cells number per crypt. Scale bar, 200 μm. **F** Endothelial tight junction protein Zo-1(red), Occludin(green) and Claudin-1(pink) were stained using immunofluorescence. Quantitative analysis of these tight junction proteins expression. Scale bar, 50 μm. Data are presented as mean ± SD. *P < 0.05, ** P < 0.01, *** P < 0.001 and ns indicates *P* > 0.05

Since the impairment of intestinal mucosal barrier is a significant characteristic of IBD (Lee 2015), we speculate that TSG-6 may inhibit the intestinal inflammatory response via improving epithelial barrier function in colitis. The permeability of the intestinal barrier was assessed via oral gavage with FITC-D, and analyzed the FITC-D fluorescence in the intestine cryosections using confocal microscopy. As shown in Fig. 3A, green fluorescence was predominantly observed within the intestinal lumen in control group, whereas in the DSS + PBS group, the majority of green fluorescence was detected in the intestinal microvilli and had infiltrated the intestinal mucosa (white arrows). Oppositely, tissue from higher dose of TSG-6 treated mice demonstrated no absorption of green fluorescence. This result was further confirmed by detecting the content of FITC-D in serum, which was notably increased in the DSS + PBS group compared with the other two groups (Fig. 3B). To further observe the intestinal barrier damage, we performed TEM to examine the ultrastructural morphology of the intestine. Decreased tight junction electron density (yellow arrows) and shortened microvilli (red arrows) were observed in PBS intervened mice on day 10 post-DSS treatment. In contrast, the higher dose of TSG-6 treatment group exhibited improved microvilli and reduced swelling of the tight junctions (Fig. 3C).

To establish the quantification of mucin-containing goblet cells, we employed PAS staining to detect both acidic and neutral mucins, which are secreted by intestinal goblet cells for the formation of a protective mucus layer crucial for host’s defense barrier. As illustrated in Fig. 3D, the DSS + H-TSG-6 group demonstrated a significantly greater count of colonic goblet cells compared to the DSS + PBS group. Since the maintenance of intestinal barrier integrity is also related to the proliferation of ISCs, we examined the expression of the proliferation marker Ki-67 in colon tissue. The result showed an increased number of Ki67-positive cells in TSG-6 treated group compared to DSS + PBS mice (Fig. 3E). Immunofluorescence was utilized to measure the distribution and expression of TJ proteins Zo-1, Occludin, and Claudin-1. All these proteins were distributed along the columnar epithelial lining, and their expression levels were significantly increased in colitis mice that received higher dose of TSG-6 treatment (Fig. 3F).

### The impact of TSG-6 proteomic changes in colon tissue

**Fig. 4:**
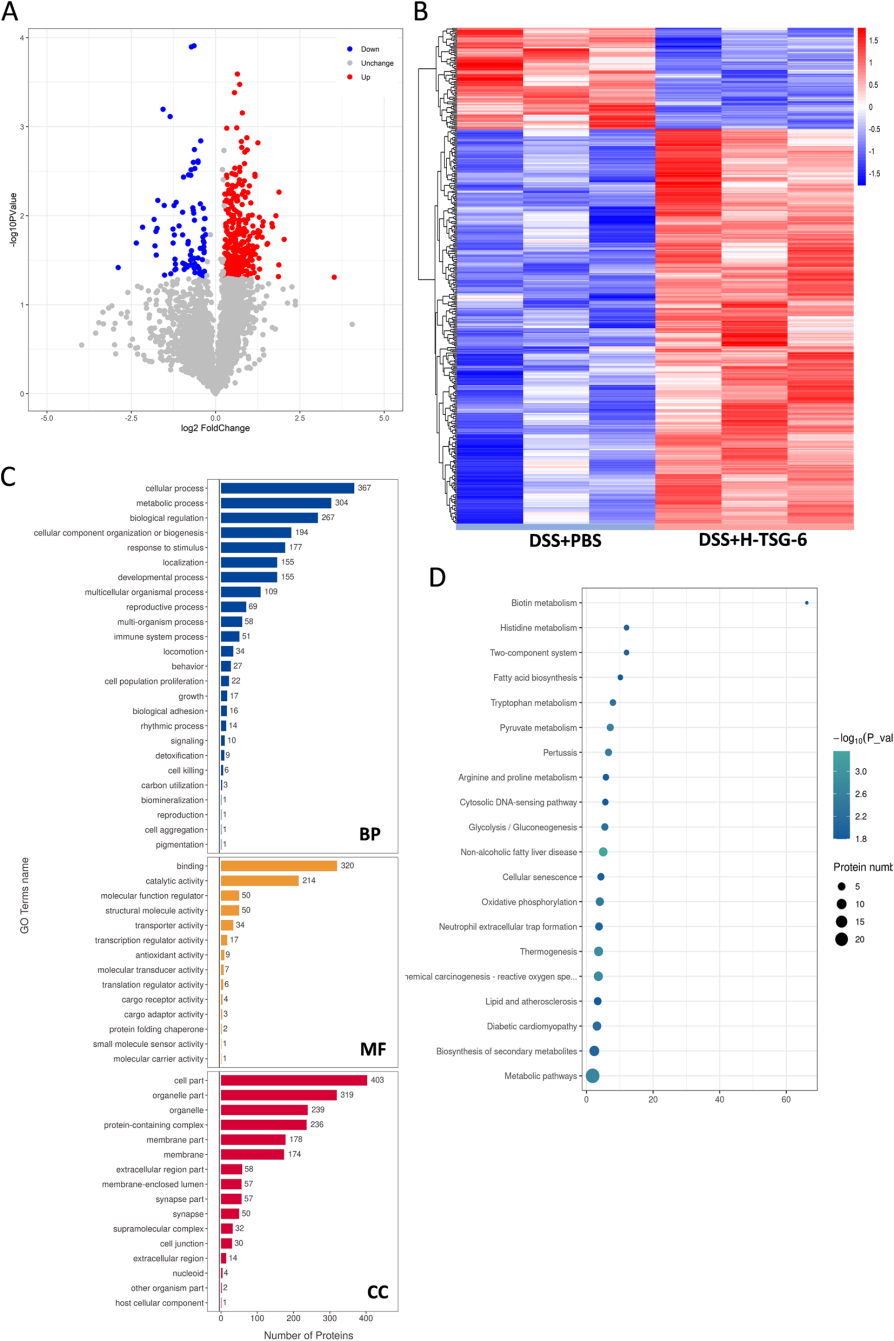
Proteomics and bioinformatics analysis are used to analyze the protein profile of TSG-6 treatment. **A** Volcano plot showing differential expressed proteins in DSS + PBS and DSS + H-TSG-6 groups. **B** Hierarchical clustering of proteins between DSS + PBS and DSS + H-TSG-6 groups. **C** Results of GO analysis of differential proteins in DSS + PBS and DSS + H-TSG-6 groups. **D** KEGG pathway analysis of differentially expressed proteins in DSS + PBS and DSS + H-TSG-6 groups

Label-free quantitative proteomic analysis was performed on intestinal samples from DSS + PBS and DSS + H-TSG-6 groups to explore the mechanism and therapeutic target of TSG-6. Herein, 5823 proteins were identified and there are 411 proteins with significantly different expressions. In comparison to the DSS + PBS group, the DSS + H-TSG-6 group exhibited an upregulation of 327 proteins and a downregulation of 84 proteins (Fig. 4A). Cluster analysis of differentially expressed proteins (DEPs) were conducted with a heatmap, exhibiting a notably different expression profile of DEPs between the two groups (Fig. 4B). The significantly enriched functional terms were analyzed by GO, including biological process (BP), molecular function (MF), and cellular component (CC) (Fig. 4C). In GO-BP, DEPs primarily engaged in the cellular process, metabolic process, and biological regulation. As for GO-MF, most of the DEPs were enriched for binding, catalytic activity, molecular function regulator and structural molecule activity. The top three terms in GO-CC included the cell part, organelle part, and organelle. Among the cellular component, we found 30 DEPs were involved in cell junction, which may be relevant to the epithelial barrier integrity. KEGG database was used to clarify the biological function of DEPs, and the pathways of top 20 were screened. As shown in Fig. 4D, key processes related to inflammatory damage and repair, such as Metabolic pathways, Chemical carcinogenesis-reactive oxygen species, Thermogenesis, Non − alcoholic fatty liver disease, and Neutrophil extracellular trap formation (NET), were closely associated with TSG-6 therapy. Our bioinformatics analysis revealed that TSG-6 likely promotes intestinal epithelial barrier integrity by modulating targeted factors involved in cell cycle, cell junctions and inflammation responses.

### TSG-6 can directly bind to Pou2f3 and upregulate its expression

**Fig. 5:**
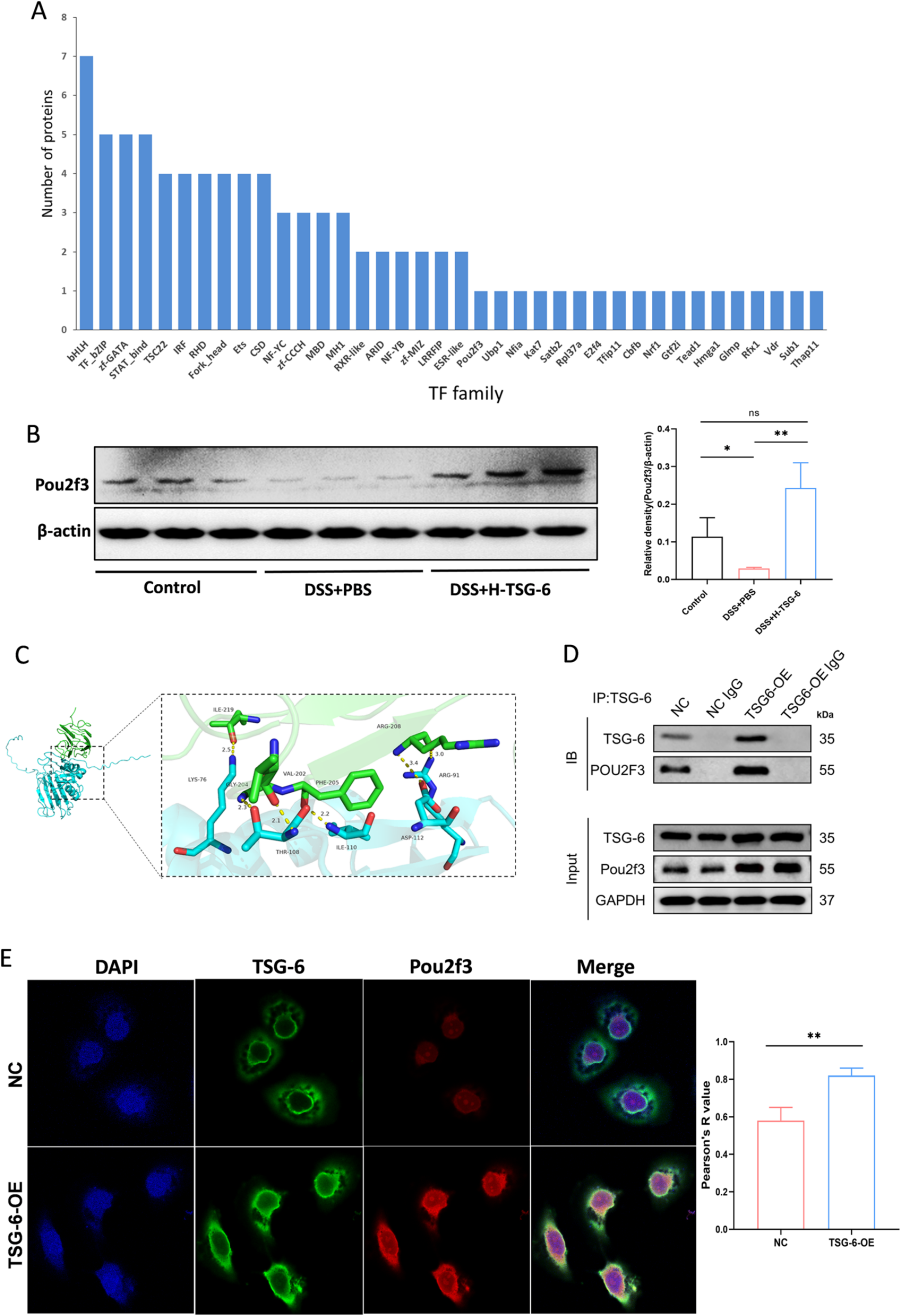
TSG-6 binds with Pou2f3 and upregulates its expression. **A** Transcription regulator binding enrichment analysis of differential expressed proteins. The 38 enriched transcription regulators are shown. **B** Quantitative analysis of the expression levels of Pou2f3 in colonic mucosa by Western blot. **C** The potential binding sites of TSG-6 against Pou2f3 displayed by molecular docking assay. **D** Co-Immunoprecipitation (Co-IP) to determine the interaction between TSG-6 and Pou2f3 in empty vector transfected (NC) and TSG-6-overpressed (OE) MODE-K cells. Co-IP was performed using mouse IgG as negative control. **E** Immunofluorescent analysis to investigate the co-localization of TSG-6(green) and Pou2f3 (red) in MODE-K cells. Statistical analysis (Pearson correlation) of TSG-6 and Pou2f3 based on fluorescence intensity. Data are presented as mean ± SD. *P < 0.05, **P < 0.01 and ns indicates *P* > 0.05

Transcription factors (TFs) perform the first step in decoding the DNA sequence, which directly interpret the genome, regulating cell differentiation and development (Lambert et al. 2018). To investigate the potential involvement of TFs and associated IEC cell types in the regulating effects of TSG-6 in colitis, we performed transcription factor annotation of DEPs by AnimalTFDB. The result shows that 38 TF families or TFs were identified to be involved in the therapeutic mechanism of TSG-6 in colitis (Fig. 5A). It is worth noting that Pou2f3, a transcription factor essential for tuft cell development, may be related to the therapeutic effect of TSG-6. To explore the role of TSG-6 in regulating the expression of Pou2f3 protein, Western blot was employed to detect Pou2f3 expression among the three groups. The results show that Pou2f3 expression was increased in DSS + H-TSG-6 group, which indicated that TSG-6 induced an upregulation of Pou2f3 protein level (Fig. 5B). To investigate the molecular interaction between TSG-6 and Pou2f3, we performed molecular docking simulations to predict their potential binding sites. Molecular docking showed that TSG-6 could directly bind with Pou2f3, and the bind may be formed and strengthened by a number of hydrogen bonds at multiple sites (Fig. 5C). Co-IP assays confirmed the interaction of TSG-6 with Pou2f3 in mouse intestinal epithelial cells (MODE-K cells), and TSG-6 overexpression significantly enhanced their interaction (Fig. 5D). Consistently, the interaction between TSG-6 and Pou2f3 was further confirmed by immunofluorescence co-localization, which also showed an increase in co-localization between TSG-6 and Pou2f3 in TSG-6-overpressed MODE-K cells (Fig. 5E).

### TSG-6 rescues Dclk1 suppression in colitis and enhances IL-25 production

**Fig. 6:**
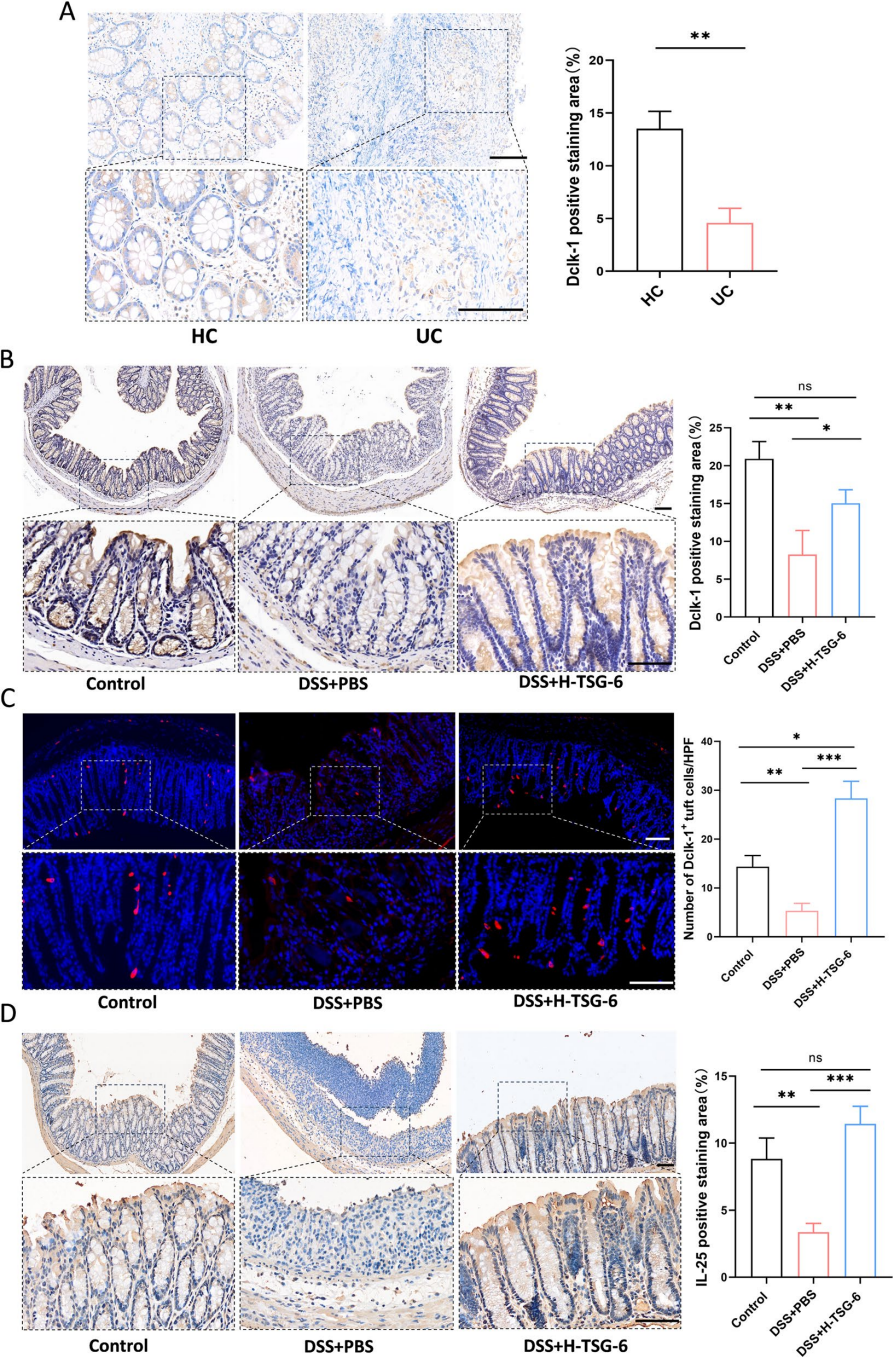
Dclk-1 is low expressed in UC and TSG-6 treatment increases the proportion of Dclk-1 + Tuft cells and the level of IL-25 in the gut. **A** Healthy control (HC) and patients with ulcerative colitis (UC) were used for Immunohistochemical staining with Dclk-1. **B** Immunohistochemical staining of Dclk-1 in mouse colon sections. Quantification of Dclk-1 positive staining area is shown in right panel. **C** Representative immunofluorescence staining of Dclk-1 positive cells and quantification. **D** Immunohistochemical staining of IL-25 in different groups. Quantification of the IL-25 positive staining area. Scale bar, 100 μm. Data are presented as mean ± SD. *P < 0.05, **P < 0.01, ***P < 0.001, and ns indicates *P* > 0.05

The data presented in Fig. 5 indicated that protein level of Pou2f3 was decreased in DSS + PBS group and could be significantly increased by TSG-6. Pou2f3 has been identified as the crucial transcription factor for the differentiation and expansion of tuft cells in infectious diseases (Zhang et al. 2018), and thus there was a strong possibility that TSG-6 could regulate the development of tuft cells by interacting with Pou2f3. To verify this hypothesis, we collected colonic mucosa samples of UC patients and healthy controls and assessed the tuft cell marker Dclk-1 expression by immunohistochemistry. The results revealed that the distribution of Dclk-1 was remarkably reduced in colonic mucosa of UC patients compared to healthy controls (Fig. 6A). In consistent with the data of clinical samples, the expression of Dclk-1 was also decreased in the colon of DSS + PBS-treated mice (Fig. 6B). We next sought to confirm if TSG-6 is sufficient to drive tuft cell differentiation in IBD mouse model and found widespread expression of Dclk-1 in DSS + H-TSG-6 group (Fig. 6B). Moreover, immunofluorescence imaging reveals consistent findings, indicating an increase in Dclk-1 + tuft cell numbers in colitis mice treated with TSG-6 (Fig. 6C). Tuft cells are the only identified source of intestinal IL-25 and initiate immune responses by secreting IL-25 (McGinty et al. 2020b; Banerjee et al. 2020). To investigate the influence of TSG-6 on the function of tuft cells, we tested the intestinal IL-25 expression and found that IL-25 expression level in the intestinal tissue of DSS + H-TSG-6 group was remarkably higher than that in the DSS + PBS group (Fig. 6D).

## Discussion

The principal objective of this research was to elucidate the therapeutic effect and mechanisms underlying TSG-6-mediated protection against intestinal epithelial barrier dysfunction in colitis. According to the experimental data, TSG-6 administration significantly reduced the release of pro-inflammatory factors and facilitated mucosal repair in murine colitis. Mechanistically, these therapeutic effects appear mediated through the upregulation of Pou2f3 expression, resulting in the promotion of colonic tuft cells expansion.

Emerging evidence positions mesenchymal stem cell (MSC) secretome-based cell-free therapy as a promising strategy in regenerative medicine (Zhao et al. 2022). As a key anti-inflammatory cytokine released from MSCs, TSG-6 mediates the therapeutic effects of MSCs in IBD through dual mechanisms of immune modulation and tissue regeneration (Zhang et al. 2021; Yang et al. 2019). While these studies suggested TSG-6's indirect therapeutic role, our work provides novel evidence that direct exogenous TSG-6 administration effectively ameliorates DSS-induced colitis through both clinical and histopathological improvements. Notably, our observation of TSG-6-induced intestinal epithelial cell (IEC) proliferation indicates a previously unrecognized association between tuft cell dynamics and TSG-6-mediated barrier restoration.

Despite the rarity of intestinal tuft cell, accounting for only 0.4% population of IECs in both the small intestine and colon, it is crucial for detecting luminal contents, thereby regulating the mucosal immune response and remodel intestinal epithelium (Xiong et al. 2021; Rosen 2020; McKinley et al. 2017). Moreover, clinical relevance is underscored by reduced tuft cell populations in CD patients and their demonstrated anti-inflammatory effects in ileitis (Banerjee et al. 2020). Pou2f3 screened by our data plays a crucial role as a transcription factor in the specialization process of tuft cells, and mice exhibit an inability to generate tuft cells following Pou2f3 knockout (DelGiorno et al. 2020). Through the prediction of molecular docking and the experimental validation of Co-IP and immunofluorescence co-localization, we established direct physical interaction between TSG-6 and Pou2f3, with subsequent elevation of Pou2f3 protein levels in IECs. The recently research showed that upregulating Pou2f3 expression could induce tuft cells expansion (Long et al. 2022). Therefore, we surmised that TSG-6 may participate in regulation of tuft cells proliferation and differentiation. To verify our hypothesis, we performed a series of in vivo assays to explore the influence of TSG-6 on tuft cells in colitis. In consistent with the changes of Pou2f3 in the IBD animal model, the expansion of Dclk-1^+^tuft cells in crypt were significantly inhibited after DSS induction, while TSG-6 treatment notably enhanced Dclk-1^+^tuft cells proliferation, which coincided with improvement of intestinal barrier function and reduction of proinflammatory mediators. These results collectively suggested that treatment with exogenous TSG-6 exerted therapeutic effects on IBD mice by increasing intestinal tuft cells, which was mediated by interaction with Pou2f3.

Besides the interaction between TSG-6 and Pou2f3, we also investigated other mechanisms by which TSG-6 may mediate IBD treatment. Label-free quantitative proteomics was used to pinpoint the proteins and metabolic pathways modulated by TSG-6. Gut microbiota-derived small-molecule metabolites are secreted into the intestinal lumen and regulated the activities of IECs, which are involved in maintain intestinal homeostasis (Postler and Ghosh 2017; Rooks and Garrett 2016). Interestingly, the KEGG enrichment analysis demonstrated that the anti-inflammatory role of TSG-6 impinged on the metabolic pathways such as pyruvate metabolism, tryptophan metabolism, and histidine metabolism, to protect against mice colitis. This aligns with emerging evidence that gut microbiota-derived metabolites (e.g., tryptophan catabolite indole-3-pyruvate) strengthen epithelial integrity through junctional complex stabilization (Scott et al. 2020). Therefore, we speculate that TSG-6 may repair the intestinal mucosal barrier by regulating metabolic pathways, which may be related to metabolites of gut microbiota. However, the current data do not establish whether TSG-6 directly regulates microbial metabolism or acts through host pathway activation. This will be a focus of future metabolomic and microbiome profiling.

According to the functional enrichment analysis, neutrophil extracellular traps (NETs) formation is another crucial enriched pathway for these DEPs. NETs are a special lattice structure produced by activated neutrophils that contain DNA, microbicidal proteins, and MPO (Papayannopoulos 2018). The antimicrobial function and defence activity of neutrophils relies on the formation of NETs. However, the aberrant NETs formation results in severe tissue damage and several immune diseases (Munir et al. 2020; Chirivi et al. 2021). Dinallo et al. (2019) found an overexpression of NETs-associated proteins in colonic tissues of patients with UC. Intestinal barrier integrity can be restored after exogenous administration of DNase I to dissolve NETs in colitis mouse model (Lin et al. 2020). Furthermore, our study indicated that TSG-6 reduced the infiltration of MPO positive neutrophils in colon tissue. Directly interfering with the NETs formation could represent a potential therapeutic approach through which TSG-6 improves intestinal inflammation. In brief, our results of proteomic provide evidence for the role of inflammatory responses and amino acid metabolism in the treatment of IBD, while the detailed mechanism in many aspects requires exploration in future work.

Despite our findings are encouraging, there are several limitations to this study need to be acknowledged. Although multiple binding sites between TSG-6 and Pou2f3 have been predicted, the precise mechanism regulating Pou2f3 expression remains undetermined. Thus, the design of Pou2f3 knockout mice therapy experiments are necessary in subsequent studies. In addition, our proteomic findings necessitate functional validation of candidate pathways. Future investigations will address these gaps through lineage-tracing studies and microbial metabolite profiling.

Taken together, our research demonstrates TSG-6 as a positive regulator of tuft cells differentiation maintaining intestinal barrier integrity to alleviate colon inflammation. Through a direct interaction with Pou2f3 and up-regulating its expression, intravenous injection of TSG-6 promotes tuft cells proliferation and increases the intestinal IL-25 content. Our research not only elucidates a therapeutic mechanism by which TSG-6 repairs the intestinal mucosa, but also provide meaningful insights for developing new preventive and therapeutic strategy for IBD.

## Supplementary Information

Supplementary Material 1.

Supplementary Material 2.

## Data Availability

No datasets were generated or analysed during the current study.
