# Supplementary material for: Exogenous TSG-6 treatment alleviates DSS-induced colitis in mice by modulating Pou2f3 and promoting tuft cells differentiation
Source: Mol Med. 2025 Apr 29;31:157. doi: 10.1186/s10020-025-01230-5 (PMC12042439; doi:10.1186/s10020-025-01230-5)
Supplement: Supplementary file 1 — Supplementary Material 1. [file 10020_2025_1230_MOESM1_ESM.docx]

**1.Label-free quantification proteomics experimental procedures**

**1.1.1 Sample Preparation**

The method was determined by the project proposal or preliminary experiment report.

i. TCA/Acetone Precipitation and SDT Lysis:

Application: Plant tissues (roots, stems ,leaves, etc）, hard tissues（skin, cartilage, hair, etc）, fungi.

The samples were frozen in liquid nitrogen and ground with a pestle and mortar. 5 times volume of TCA/acetone (1:9) was added to the powder and mixed by vortex. The mixture was placed at -20°C for 4h, and centrifuged at 6000g for 40 min at 4°C. The supernatant was discarded. The pre-cooling acetone was added and washed for three times. The precipitation was air dried. 30 times volume of SDT buffer (4%SDS，100mM Tris-HCl，pH 7.6) was added to 20-30 mg powder, mixed and boiled for 5 min. The lysate was sonicated and then boiled for 15 min. After centrifuged at 14000g for 15 min, the supernatant was filtered with 0.22 µm filters. The filtrate was quantified with the BCA Protein Assay Kit (P0012, Beyotime). The sample was stored at -80 °C.

ii. Homogenate and SDT Lysis：

Application: Tender tissues (brain, liver, muscle, etc), mollusk, microorganism, etc.

SDT buffer was added to the sample, and transferred to 2 ml tubes with amount quartz sand. The lysate was homogenized by MP Fastprep-24 Automated Homogenizer (6.0M/S, 30s, twice). The homogenate was sonicated and then boiled for 10 min. After centrifuged at 14000g for 15 min, the supernatant was filtered with 0.22 µm filters. The filtrate was quantified with the BCA Protein Assay Kit (P0012, Beyotime). The sample was stored at -80 °C

iii. SDT Lysis：

Application：Cell, protein powder, body fluid, concentrated fermentation broth, cell secretion, etc.

SDT buffer (4%SDS，100mM Tris-HCl，pH 7.6) was added to the sample. The lysate was sonicated (this step can be skipped for protein solution) and then boiled for 15 min. After centrifuged at 14000g for 15 min, the supernatant was quantified with the BCA Protein Assay Kit (P0012, Beyotime). The sample was stored at -80 °C.

iv. Immunoaffinity Depletion of Serum High-Abundance Proteins：

Application： human, mouse or rat serum

Serum pools were depleted of most abundant proteins using Agilent Human 14 / Mouse 3 Multiple Affinity Removal System Column following the manufacturer’s protocol [10-12] (Agilent Technologies). The Human 14 column was applied for human, and Mouse 3 column was applied for mouse and rat. The 15 kDa ultrafiltration tube (Sartorius) was used for desalination and concentration of low-abundance components. One volume of SDT buffer (4%SDS，100mM Tris-HCl，pH 7.6) was added, boiled for 10min and centrifuged at 14000g for 15 min. The supernatant was quantified with the BCA Protein Assay Kit (Bio-Rad, USA). The sample was stored at -80 °C.

5.1.2 SDS-PAGE Separation

20 µg of proteins for each sample were mixed with 6X loading buffer respectively and boiled for 5 min. The proteins were separated on 12% SDS-PAGE gel. Protein bands were visualized by Coomassie Blue R-250 staining.

5.1.3 Filter-aided sample preparation (FASP Digestion)

50-200 μg of proteins for each sample were reduced with 100 mM DTT for 5 min at 100 °C. Then the detergent, DTT and other low-molecular-weight components were removed using UA buffer (8 M Urea, 150 mM Tris-HCl pH 8.5) by repeated ultrafiltration (Sartorius, 30 kD). Then 100 μl iodoacetamide (100 mM IAA in UA buffer) was added to block reduced cysteine residues and the samples were incubated for 30 min in darkness. The filters were washed with 100 μl UA buffer three times and then 100 μl 50 mM NH4HCO3 buffer twice.Finally, the protein suspensions were digested with 4 μg trypsin (Promega) in 40 μl 50 mM NH4HCO3 buffer overnight at 37 °C, and the resulting peptides were collected as a filtrate. The peptide segment was desalted by C18 column. The peptide content was estimated by UV light spectral density at 280 nm using an extinctions coefficient of 1.1 of 0.1% (g/l) solution that was calculated on the basis of the frequency of tryptophan and tyrosine in vertebrate proteins.

5.2 Mass Spectrometry analysis

The peptide of each sample was desalted on C18 Cartridges, then concentrated by vacuum centrifugation and reconstituted in 40 µl of 0.1% (v/v) formic acid. The peptide content was estimated by UV light spectral density at 280 nm using an extinctions coefficient of 1.1 of 0.1% (g/l) solution that was calculated on the basis of the frequency of tryptophan and tyrosine in vertebrate proteins. LC-MS/MS analysis was performed on a Orbitrap Exploris 480 mass spectrometer (Thermo Fisher Scientific) that was coupled to Easy nLC (Thermo Fisher Scientific). 2μg peptide was loaded onto the C18-reversed phase analytical column (Thermo Fisher Scientific，Acclaim PepMap RSLC 50um X 15cm, nano viper,P/N164943) in buffer A (0.1% formic acid) and separated with a linear gradient of buffer B (80% acetonitrile and 0.1% Formic acid) at a flow rate of 300 nl/min.

MS data was acquired using a data-dependent top10 method dynamically choosing the most abundant precursor ions from the survey scan (350–1200 m/z) for HCD fragmentation. MS1 scans were acquired at a resolution of 120000 at m/z 200 with an AGC target of 300% and a max IT of 50 ms. Data dependent mode is cycle time, and cycle time is set to 1.5s.MS2 scans were acquired at a resolution of 15000 at m/z 200 with an AGC target of 75% and a max IT of 35 ms, and isolation width was 1.6 m/z. Microscans set to 1.Only ions with a charge state between 2-6. Dynamic exclusion for selected ions was 30s. Normalized collision energy was 33%.

**2.Bioinformatics analysis**

**2.1 Gene Ontology (GO) Annotation**

At first, all protein sequences were aligned to the database ( determined by project ) using NCBI BLAST+（ ncbi-blast-2.3.0+ ）on the Linux server, only the sequences in top 10 and E-value<=1e-3 were kept. Secondly, select the GO term (database version: go_20190701.obo ) of the sequence with top Bit-Score by Blast2GO. Then, completed the annotation from GO terms to proteins by Blast2GO Command Line. After the elementary annotation, InterProScan were used to search EBI database by motif and then add the functional information of motif to proteins to improve annotation. Then further improvement of annotation and connection between GO terms were carried out by ANNEX. Fisher’s Exact Test were used to enrich GO terms by comparing the number of differentially expressed proteins and total proteins correlated to GO terms.

**2.2 KEGG Pathway Annotation**

Pathway analysis was performed using KEGG database (database version: KO_INFO_END.txt ( 2023.03.24 ) . Fisher's Exact Test were used to identify the significantly enriched pathways by comparing the number of differentially expressed proteins and total proteins correlated to pathways.

**3. Cell culture and transfection**

Mouse intestinal epithelial cells (MODE-K) was purchased from XiaoYan Biotechnology (Shanghai, China). The cells were cultured in Dulbecco’s modified eagle’s medium (DMEM) (Gibco) containing 10% fetal bovine serum (FBS, Gibco) at 37°C in 5% CO_2_. For overexpression, full-length TSG-6 cDNA was cloned into lentiviral expressing vector pLV-puro. Production of lentiviral particles was performed according to the standard protocols. Cells were transfected with lentiviral constructs expressing empty vector (NC) or TSG-6 (TSG-6-OE) for 48 hours with 5 μg/ml polybrene (Sigma). After 48 hours, stable cells were selected by using 1 μg/ml puromycin for 7 days.

**4. Co-immunoprecipitation (co-IP)**

MODE-K cells transfected with TSG-6 or Vector for 48 hours were lysed in 600μl IP lysis buffer for 30 minutes on ice. Cell lysates were centrifugated at 12,000g for 10 minutes at 4℃ and the protein supernatant was collected, 60 ul supernatant was used as input. Then the remaining protein supernatant was incubated with Mouse IgG1 Isotype Control (R&D System, MAB002) or Anti-Pou2f3 (Novus, 81774) overnight at 4℃. Then the mixture was incubated with the Sepharose-conjugated protein G magnetic beads (Thermo Fisher Scientific, MA, USA) overnight at 4℃. After washing with IP wash buffer for four times, the beads were centrifugated and mixed with 1x loading buffer and boiled for 10 minutes. The protein of co-IP and input samples were determined by western blot analysis.

**5. Cell Immunofluorescence**

MODE-K cells were harvested after transfection with lentiviral constructs expressing empty vector or TSG-6 for 48 hours, and then plated on the coverslips. For staining of TSG-6 and Pou2f3, cells were fixed with 4% paraformaldehyde for 20 minutes at room temperature, permeabilized with 0.1% Triton X-100 for 5 minutes, then blocked with 1% bovine serum albumin (BSA) for 45 minutes. Then cells were incubated simultaneously with both anti-TSG-6 (1:200) and anti-Pou2f3 (1:100) at 4℃ overnight. The following day, cells were simultaneously incubated with both goat anti-rabbit Alexa Fluor 488 conjugated secondary antibody and goat anti-mouse Alexa Fluor 542 conjugated secondary antibody (Invitrogen, USA) at room temperature for 1 hour, followed by staining cell nuclei with DAPI (Sigma Aldrich, Germany). The images were captured by Leica TCS-SP8 confocal microscopy (Mannheim, Germany).

**6. Myeloperoxidase (MPO) activity assay**

Infiltration of neutrophils into colonic tissue was quantified by MPO activity measurement with an MPO assay kit (Nanjing Jiancheng Bio-engineering Institute, Nanjing, China) according to the manufacturer’s instructions. MPO activity was expressed as units per gram of total protein (U/g).

**7. Table S1 The demography of the human sample**

**8. Table S2 Scoring system for calculating a disease activity index (DAI)**

| Score | Weight  loss | Stool  consistency | Blood |
| --- | --- | --- | --- |
| 0 | None | Norma | Negative hemocult |
| 1 | 1–5% | Soft but still formed | Negative hemocult |
| 2 | 6–10% | Soft | Positive hemocult |
| 3 | 11–18% | Very soft; wet | Blood traces in stool visible |
| 4 | >18% | Watery diarrhea | Gross rectal bleeding |

**9. Table S3 Histopathological Scoring Criteria for DSS-Induced Colitis in Mice**

| **Histological Feature** | **Score** | **Description** |
| --- | --- | --- |
| **Inflammation (I)** |  |  |
|  | 0 | None |
|  | 1 | Mild (scattered inflammatory cells in lamina propria) |
|  | 2 | Moderate (inflammatory cells extending into submucosa) |
|  | 3 | Severe (transmural inflammation) |
| **Depth (E)** |  |  |
|  | 0 | None |
|  | 1 | Mucosal layer only |
|  | 2 | Mucosa and submucosa |
|  | 3 | Transmural (through all layers) |
| **Regeneration (R)** |  |  |
|  | 0 | No tissue repair |
|  | 1 | Incomplete regeneration (partial epithelial restoration) |
|  | 2 | Almost complete regeneration (near-normal crypt architecture) |
|  | 3 | Regeneration with crypt loss (abnormal crypt structure) |
| **Crypt Damage (C)** |  |  |
|  | 0 | None |
|  | 1 | Basal 1/3 crypt destruction |
|  | 2 | Basal 2/3 crypt destruction |
|  | 3 | Only surface epithelium intact |
|  | 4 | Complete crypt and epithelial loss |
| **Percentage Involvement (P)** |  |  |
|  | 1 | 1%-25% of tissue affected |
|  | 2 | 26%-50% of tissue affected |
|  | 3 | 51%-75% of tissue affected |
|  | 4 | 76%-100% of tissue affected |

**Total Histological Score** = **I** (Inflammation) + **E** (Depth) + **R** (Regeneration) + **C** (Crypt Damage) + **P** (Percentage Involvement)
**Score Range**: 0–17 (higher scores indicate more severe pathology).
