# Supplementary figures and images for: Exogenous TSG-6 treatment alleviates DSS-induced colitis in mice by modulating Pou2f3 and promoting tuft cells differentiation
Source: Mol Med. 2025 Apr 29;31:157. doi: 10.1186/s10020-025-01230-5 (PMC12042439; doi:10.1186/s10020-025-01230-5)

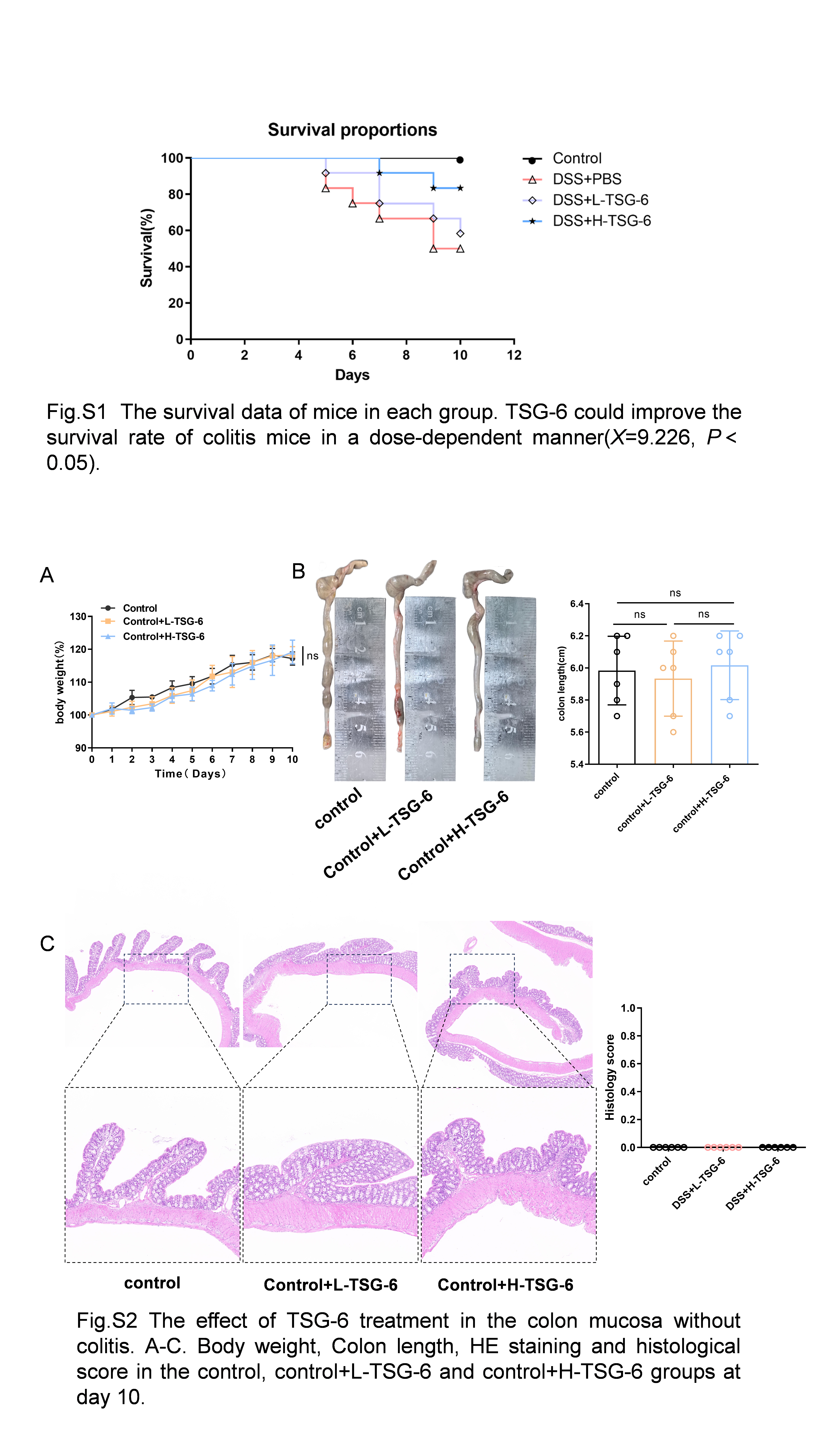

Supplement: Supplementary file 2 — Supplementary Material 2. [file 10020_2025_1230_MOESM2_ESM.tif]
